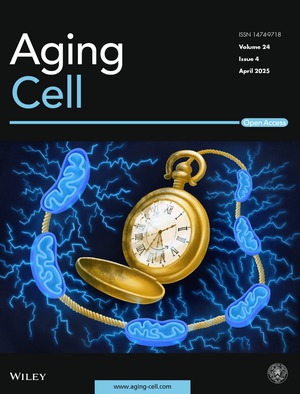# Additional Cover

**DOI:** 10.1111/acel.70073

**Published:** 2025-04-10

**Authors:** Hyunho Lee, Matteo Massaro, Nourhan Abdelfattah, Gherardo Baudo, Haoran Liu, Kyuson Yun, Elvin Blanco

## Abstract

The cover image is based on the article *Nuclear respiratory factor‐1 (NRF1) induction as a powerful strategy to deter mitochondrial dysfunction and senescence in mesenchymal stem cells* by Hyunho Lee et al., https://doi.org/10.1111/acel.14446.